# Colesevelam ameliorates non-alcoholic steatohepatitis and obesity in mice

**DOI:** 10.1007/s12072-022-10296-w

**Published:** 2022-01-24

**Authors:** Phillipp Hartmann, Yi Duan, Yukiko Miyamoto, Münevver Demir, Sonja Lang, Elda Hasa, Patrick Stern, Dennis Yamashita, Mary Conrad, Lars Eckmann, Bernd Schnabl

**Affiliations:** 1 Department of Pediatrics, University of California San Diego, La Jolla, CA USA; 2 Department of Medicine, University of California San Diego, MC0063, 9500 Gilman Drive, La Jolla, CA 92093 USA; 3 Department of Hepatology and Gastroenterology, Campus Virchow Clinic and Campus Charité Mitte, Charité University Medicine, Berlin, Germany; 4 Faculty of Medicine, and University Hospital Cologne, Department of Gastroenterology and Hepatology, University of Cologne, Cologne, Germany; 5 Axial Biotherapeutics, Woburn, MA USA; 6 Department of Medicine, VA San Diego Healthcare System, San Diego, CA USA

**Keywords:** Bile acid sequestrants, Humanized mice, Non-alcoholic fatty liver disease, Experimental liver disease, Liver inflammation, Liver steatosis, Liver fibrosis, Insulin resistance, Western diet, Rodents

## Abstract

**Background:**

Obesity, non-alcoholic fatty liver disease (NAFLD) and its more advanced form non-alcoholic steatohepatitis (NASH) are important causes of morbidity and mortality worldwide. Bile acid dysregulation is a pivotal part in their pathogenesis. The aim of this study was to evaluate the bile acid sequestrant colesevelam in a microbiome-humanized mouse model of diet-induced obesity and steatohepatitis.

**Methods:**

Germ-free C57BL/6 mice were associated with stool from patients with NASH and subjected to 20 weeks of Western diet feeding with and without colesevelam.

**Results:**

Colesevelam reduced Western diet-induced body and liver weight gain in microbiome-humanized mice compared with controls. It ameliorated Western diet-induced hepatic inflammation, steatosis, fibrosis and insulin resistance. Colesevelam increased de novo bile acid synthesis and decreased hepatic cholesterol content in microbiome-humanized mice fed a Western diet. It further induced the gene expression of the antimicrobials *Reg3g* and *Reg3b* in the distal small intestine and decreased plasma levels of LPS.

**Conclusions:**

Colesevelam ameliorates Western diet-induced steatohepatitis and obesity in microbiome-humanized mice.

## Introduction

The prevalence of obesity (body mass index ≥ 30 kg/m^2^) is 19.5% in countries of the Organization for Economic Cooperation and Development [1]. One out of 4 subjects suffers from non-alcoholic fatty liver disease (NAFLD) globally [2]. Non-alcoholic steatohepatitis (NASH), a more advanced and serious form of NAFLD, is now the second most common indication for liver transplantation in the United States after alcohol-associated liver disease [2–4]. Lifestyle modifications, including a well-balanced diet and daily physical exercise, are the primary treatment options for obesity, NAFLD and NASH [5]. Despite these lifestyle modifications, obesity and NAFLD/NASH oftentimes progress. Effective pharmacologic treatment options are currently lacking.

Bile acid sequestrants bind bile acids in the intestinal lumen, thereby inhibiting their enterohepatic circulation, and induce de novo synthesis of bile acids from cholesterol in the liver [6, 7]. The bile acid sequestrant colesevelam, when administered orally, ameliorates bile acid malabsorption-associated diarrhea in patients with Crohn's disease [8] and reduces hemoglobin A1c and low-density lipoprotein-cholesterol in patients with type 2 diabetes mellitus [9, 10].

The aim of this study was to evaluate the effects of colesevelam in a microbiome-humanized mouse model of Western diet-induced steatohepatitis and obesity.

## Material and methods

### Mice

Male C57BL/6 germ-free mice were bred at the University of California San Diego (UCSD). Fecal microbiota transplantation with non-pooled samples from 2 patients with non-alcoholic steatohepatitis (NASH) (Table 1) was performed at the age of 5–6 weeks and repeated 2 weeks later as described [11]. Briefly, mice were gavaged with 100 μL of a stool suspension, which was prepared by dissolving 1 g stool in 30 mL Luria–Bertani (LB) medium containing 15% glycerol under anaerobic conditions. Two weeks after the second gavage, mice were placed for 20 weeks on an irradiated Western‐style fast‐food diet (“Western diet”) (TD.200289; containing 41.9% kcal from fat, 43.0% kcal from carbohydrate, 15.2% from protein, 4.6 kcal/g) with or without 2% colesevelam (obtained from Apothecon Pharmaceuticals). As a control, mice were fed for 20 weeks with an irradiated low-fat control diet (“chow diet”) (TD.110637; containing 13.0% kcal from fat, 67.9% kcal from carbohydrate, 19.1% kcal from protein, 3.6 kcal/g) with or without 2% colesevelam. Diets were manufactured by Teklad Diets, Madison, WI. We have replenished the diet every 2 weeks and quantitated the daily amount of diet consumed by dividing the amount of diet consumed over the 2 weeks by 14 and by the respective weight of the mice at the time of replenishment. The areas under the curve of diet consumption per body weight were used to determine differences between the groups. For the subcutaneous adipose tissue measurement, the white adipose tissue between the skin and the peritoneum in the lower abdomen below the rib cage was quantitated after an anterior-median abdominal dissection through the skin and subsequent dissection through the skin in the transverse plane below the rib cage. For the mesenteric adipose tissue measurement, the white adipose tissue located between stomach, intestine, pancreas, and spleen was quantitated. Mice were randomly assigned to the different groups at the beginning of the study. Animals were maintained on a 12 h:12 h light–dark cycle in Sentry SPP systems (Allentown, NJ) under gnotobiotic conditions [12]. All manipulations were performed during the light cycle.

**Table 1:** Characteristics of patients with NASH used as stool donors for germ-free mice

Variables	NASH	
	Patient 1	Patient 2
*Demographic*s		
Gender	Female	Female
Body mass index, kg/m^2^	31.6	30.9
Type 2 diabetes	Yes	No
Arterial hypertension	Yes	Yes
Metabolic syndrome	Yes	Yes
Waist circumference (cm)	115	100
*Laboratory parameters*		
Albumin, g/dL	4.0	4.2
Creatinine, mg/dL	1.28	1.03
Urea, mg/dL	39	31
Uric acid, mg/dL	8.4	7.8
AST, U/L	83	38
ALT, U/L	78	43
GGT, U/L	334	86
Alkaline phosphatase, U/L	98	103
Bilirubin, mg/dL	0.4	0.4
Ferritin, μg/L	94	93
Triglycerides, mg/dL	384	231
Total cholesterol, mg/dL	206	251
HDL cholesterol mg/dL	33	38
LDL cholesterol mg/dL	114	156
Platelet count, × 10^9^/L	319	261
INR	2.4	0.9
*Liver histology features*^&^		
NAFLD Activity Score	5	7
Steatosis grade	1	3
Ballooning	2	2
Inflammation grade	2	2
Fibrosis stage	3	2

^&^per NASH clinical research network histologic scoring system [29]. *ALP *alkaline phosphatase,* ALT *alanine aminotransferase,* AST *aspartate aminotransferase,* GGT *gamma-glutamyl transferase,* HDL *high-density lipoprotein, *INR *international normalized ratio,* LDL *low-density lipoprotein,* NAFLD *non-alcoholic fatty liver disease, *NASH *non-alcoholic steatohepatitis

After 16 weeks of feeding, an insulin tolerance test was performed by injecting insulin (Novolin N NPH; Novo Nordisk Inc., Princeton, NJ) at a dose of 1 mU/g body weight intraperitoneally. Prior to the insulin tolerance test, mice were fasted for 10 h. Tail vein blood for measuring blood glucose levels was obtained before injection (*t* = 0 min) and at *t* = 30, 60, 90, 120 min after insulin injection. The areas under the curve of the blood glucose levels were used to determine differences between the groups.

### Real-time quantitative PCR

RNA was extracted from liver or distal small intestinal tissue using Trizol (Invitrogen). RNA was digested with DNase using the DNA-free DNA removal kit (Ambion) and cDNAs were generated using the high-capacity cDNA revere transcription kit (Applied Biosystems). All primer sequences for mouse genes (18S, tumor necrosis factor-alpha (Tnf-α), chemokine C–C motif ligand-2 (Ccl-2), adhesion G protein-coupled receptor E1 (Adgre1; also known as F4/80), transforming growth factor-beta 1 (Tgfb1), tissue inhibitor of metalloproteinase-1 (Timp1), collagen type I alpha 1 (Col1a1), cytochrome P450 7a1 (Cyp7a1), 8b1 (Cyp8b1), 27a1 (Cyp27a1), 7b1 (Cyp7b1), occludin, claudin 1, claudin 4, small heterodimer partner (Shp), fibroblast growth factor 15 (Fgf15), regenerating islet-derived protein 3 gamma and beta (Reg3g and Reg3b)) were obtained from the NIH qPrimerDepot and are listed in Table 2. Gene expression was determined by quantitative PCR using Sybr Green (Bio-Rad Laboratories) and an ABI StepOnePlus real-time PCR system. Gene expression was normalized relative to 18S RNA levels.

**Table 2:** List of quantitative PCR primers and their sequence

Name	Forward	Reverse
18S	AGTCCCTGCCCTTTGTACACA	CGATCCGAGGGCCTCACTA
Tnfa	AGGGTCTGGGCCATAGAACT	CCACCACGCTCTTCTGTCTAC
Ccl2	ATTGGGATCATCTTGCTGGT	CCTGCTGTTCACAGTTGCC
Adgre1/F4/80	GGATGTACAGATGGGGGATG	CATAAGCTGGGCAAGTGGTA
Tgfb1	GGAGAGCCCTGGATACCAAC	CAACCCAGGTCCTTCCTAAA
Timp1	AGGTGGTCTCGTTGATTTCT	GTAAGGCCTGTAGCTGTGCC
Col1a1	TAGGCCATTGTGTATGCAGC	ACATGTTCAGCTTTGTGGACC
Cyp7a1	GGGAATGCCATTTACTTGGA	GTCCGGATATTCAAGGATGC
Cyp8b1	TCCTCAGGGTGGTACAGGAG	GATAGGGGAAGAGAGCCACC
Cyp27a1	CTATGTGCTGCACTTGCCC	ACTTGCCCTCCTGTCTCATC
Cyp7b1	TCCTAGGCCTTCTCTTTGCC	TTATCAAGGGTGGTTCACGA
Occludin	CATAGTCAGATGGGGGTGGA	ATTTATGATGAACAGCCCCC
Claudin 1	ATGCCAATTACCATCAAGGC	GAGGGACTGTGGATGTCCTG
Claudin 4	AGCAAACGTCCACTGTCCTT	CCCTCATCAGTCACTCAGCA
Shp	AAGACTTCACACAGTGCCCA	CACGATCCTCTTCAACCCAG
Fgf15	GAGGACCAAAACGAACGAAATT	ACGTCCTTGATGGCAATCG
Reg3g	AAGCTTCCTTCCTGTCCTCC	TCCACCTCTGTTGGGTTCAT
Reg3b	GGCTTCATTCTTGTCCTCCA	TCCACCTCCATTGGGTTCT

### Histological staining procedures

Formalin-fixed tissue samples were embedded in paraffin (Paraplast plus, McCornick), 5 μm frozen sections were cut and stained with hematoxylin–eosin (Surgipath) or 0.1% picrosirius red (color index 35,780, 365,548; Sigma‐Aldrich). To determine lipid accumulation, liver sections were embedded in OCT compound, and 10 μm frozen sections were cut and stained with Oil Red O (Sigma-Aldrich).

### Biochemical analysis

Hepatic triglyceride levels were measured using the Triglyceride Liquid Reagents Kit (Pointe Scientific). For fecal triglyceride quantitation, fecal samples were mixed with phosphate-buffered saline (PBS) in a 1:40 dilution and centrifuged at 12,000 g for 5 min to separate solid particles. Triglyceride concentrations in supernatants were determined as previously described employing the Triglyceride Liquid Reagents Kit (Pointe Scientific) [13]. Hepatic cholesterol levels were measured using the Cholesterol Liquid Reagents Kit (Pointe Scientific). Total serum bile acids were measured using a Mouse Total Bile Acid kit (Crystal Chem). Plasma lipopolysaccharide (LPS) was quantitated using an ELISA Kit for Lipopolysaccharides (LPS) (Lifeome Biolabs). For determination of tissue hydroxyproline content, liver specimens (100 mg) were homogenized in 6 N HCl (3750‐32; USABlueBook), using lysing matrix C tubes with the Mini‐BeadBeater‐96, and subsequently incubated at 110 °C for 24 h. The lysate was filtered using Whatman filter paper, grade 595 1/2 (WHA10311644; Sigma-Aldrich). After incubation with chloramine T– (C9887; Sigma-Aldrich) and Ehrlich's perchloric acid solution (AC168760250; Thermo Fisher Scientific), samples were measured at 558 nm (VersaMax Microplate Reader; Molecular Devices LLC, Sunnyvale, CA).

### Statistical analysis

Results are expressed as mean ± s.e.m. Numbers for biological replicates are *n* = 5 for chow diet and *n* = 15 for Western diet groups, unless denoted differently in the figure legends. Two technical replicates were performed in the chow diet-fed group and six technical replicates in the Western diet-fed group. Significance was evaluated using One- or Two-way analysis of variance (ANOVA) with Holm–Šídák’s post hoc test, or unpaired student *t* test, as indicated, if the values passed the Kolmogorov–Smirnov normality test. If the Kolmogorov–Smirnov normality test could not be calculated due to small *n* or if it failed, the Mann–Whitney test was employed to determine significance. A *p* value < 0.05 was considered to be statistically significant. Statistical analyses were performed using R statistical software, R version 1.3.1056 for Mac, 2020 the R Foundation for Statistical Computing, and GraphPad Prism 8.4.0 for Mac.

## Results

### Colesevelam improves Western diet-induced obesity and liver weight gain in microbiome-humanized mice

**Fig. 1:**
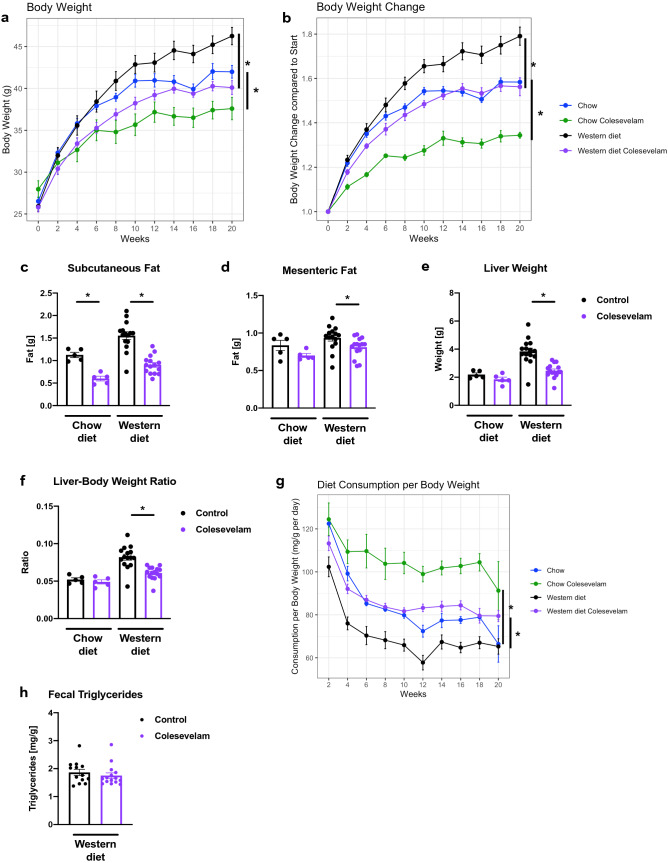
Colesevelam decreases Western diet-induced body and liver weight gain despite increased food consumption in microbiome-humanized mice. Germ-free C57BL/6 mice were colonized with feces from two patients with NASH and subjected to 20 weeks of Western diet-feeding with or without colesevelam (*n* = 13–15 per group) or chow diet with or without colesevelam (*n* = 5 per group). **a** Absolute body weight. **b** Body weight gain relative to start of feeding experiment. **c** Weight of subcutaneous white adipose tissue. **d** Weight of mesenteric white adipose tissue. **e** Liver weight. **f** Liver weight-to-body weight ratio. **g** Daily food consumption in mg per g body weight. **h** Fecal triglyceride content. Results expressed as mean ± s.e.m. *p* values are determined by one-way ANOVA with Holm’s post hoc test (**a**–**g**) or Mann–Whitney test (**h**). *p < 0.05. *NASH*, non-alcoholic steatohepatitis

Germ-free C57BL/6 mice were associated with fecal microbiota from two patients with NASH (Table 1) and subjected to feeding of a Western diet over 20 weeks. Colesevelam treatment resulted in lower absolute body weight and reduced body weight gain compared with Western diet-fed control mice (Fig. 1A–B). Administration of the bile acid sequestrant decreased subcutaneous and mesenteric white fat gain in relation to controls (Fig. 1C–D). Further, liver weight and liver weight-to-body weight ratio were decreased in Western diet-fed mice receiving colesevelam relative to Western diet-fed control mice (Fig. 1E–F). These changes occurred despite increased food intake of the Western diet in colesevelam-treated mice versus their controls (Fig. 1G). The fecal triglyceride content was not significantly different between the Western diet-fed mouse groups (Fig. 1H). Colesevelam did not result in apparent loose stools in the mice.

### Colesevelam ameliorates Western diet-induced hepatic inflammation, steatosis, and insulin resistance in microbiome-humanized mice

**Fig. 2:**
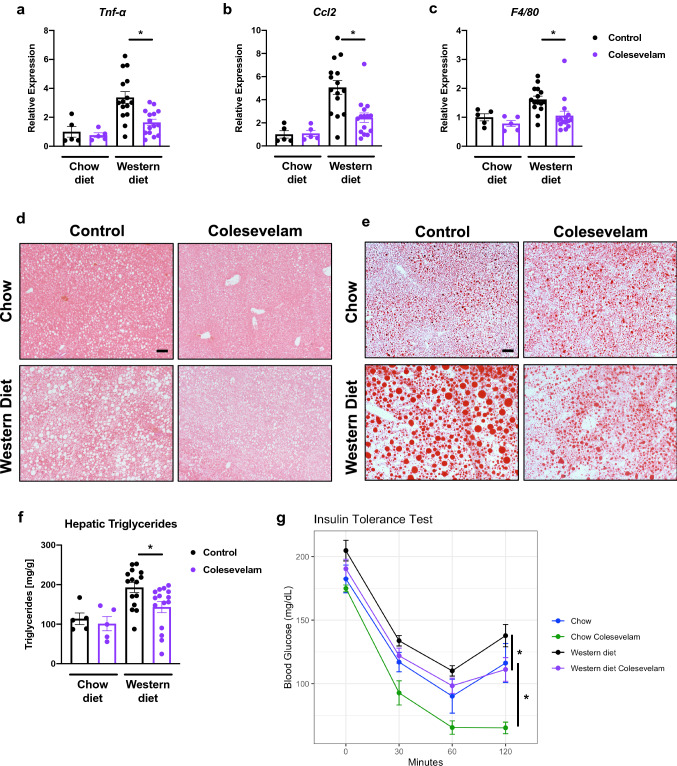
Colesevelam decreases Western diet-induced hepatic inflammation and steatosis, and improves insulin sensitivity in microbiome-humanized mice. Microbiome-humanized C57BL/6 mice were fed a chow diet (*n* = 4–8) or Western diet (*n* = 11–24) with or without colesevelam for 20 weeks. **a–c** Hepatic mRNA expression of **a** *Tnf-α*, **b** *Ccl2*, and **c** *F4/80*. **d**–**e** Representative liver sections after (**d**) hematoxylin and eosin staining (bar size = 100 $\mu$m) and (**e**) after Oil Red O staining (bar size = 100 $\mu$m). **f** Hepatic triglyceride content. **G** Insulin tolerance test. Results expressed as mean ± s.e.m. P values are determined by One-way ANOVA with Holm’s post hoc test (**a**–**b**) or Mann–Whitney test (**c**, **f**–**g**). *p < 0.05. *Ccl2,* chemokine C–C motif ligand-2; *Tnf-α*, tumor necrosis factor-alpha

Colesevelam reduced hepatic inflammation as evidenced by lower hepatic mRNA expression of tumor necrosis factor-alpha (*Tnf-α),* chemokine C–C motif ligand-2 (*Ccl2*), and adhesion G protein-coupled receptor E1 (*Adgre1*), also known as *F4/80*, (Fig. 2A–C) in comparison with Western diet-fed controls. Additionally, it reduced liver steatosis in Western diet-fed mice, as indicated by significantly lower hepatic triglycerides and fewer and smaller fat droplets on staining in relation to Western diet-fed mice not administered the bile acid sequestrant (Fig. 2D–F). Colesevelam also improved insulin sensitivity in Western diet-fed mice, as determined by insulin tolerance test (Fig. 2G).

### Colesevelam reduces Western diet-induced hepatic fibrosis in microbiome-humanized mice

**Fig. 3:**
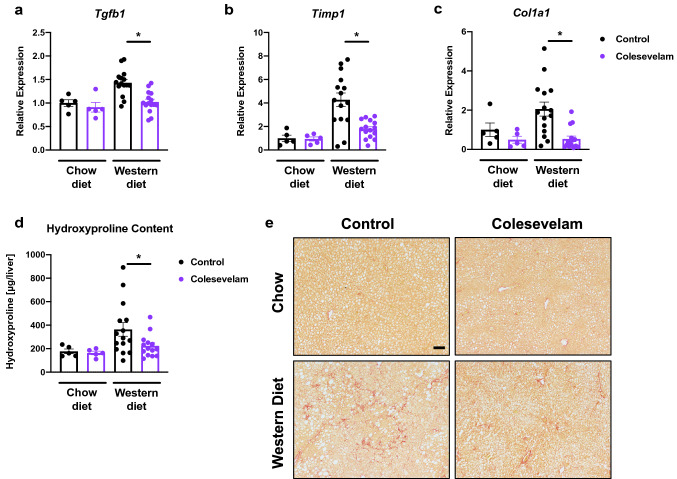
Colesevelam decreases Western diet-induced hepatic fibrosis in microbiome-humanized mice. Microbiome-humanized C57BL/6 mice were fed a chow diet (*n* = 5) or Western diet (*n* = 14–15) with or without colesevelam for 20 weeks. **a**–**c** Hepatic mRNA expression of **a** *Tgfb1*, **b** *Timp1*, and **c** *Col1a1*. **d** Hepatic hydroxyproline content. **e** Representative liver sections after Sirius Red staining (bar size = 100 $\mu$m). Results expressed as mean ± s.e.m. *p* values are determined by Mann–Whitney test (**a**, **c**) or One-way ANOVA with Holm’s post-hoc test (**b**, **d**). *p < 0.05. *Col1a1*, collagen type I alpha 1; *Tgfb1*, transforming growth factor-beta 1; *Timp1*, tissue inhibitor of metalloproteinase-1

Twenty weeks of Western diet feeding resulted in liver fibrosis in microbiome-humanized mice, as determined by increased hepatic mRNA expression of genes involved in fibrosis, including transforming growth factor-beta 1 (*Tgfb1*), tissue inhibitor of metalloproteinase-1 (*Timp1*), and collagen type I alpha 1 (*Col1a1*) (Fig. 3A–C). These changes were attenuated when mice were treated with colesevelam. Reduced liver fibrosis was confirmed by reduced total hepatic hydroxyproline content and by Sirius Red staining of liver sections in Western diet-fed mice relative to their Western diet-fed counterparts (Fig. 3D–E). A total of two patients with NASH were chosen as stool donors. One of the NASH patients had type 2 diabetes mellitus; however, we did not observe significant differences in body weight and liver disease markers such as liver weight-to-body weight ratio, hepatic triglycerides, gene expression of inflammatory and fibrosis markers including *Tnf-α* and *Tgfb1*, respectively, between mice that received stool from the NASH patient with type 2 diabetes mellitus and mice that received stool from the NASH patient without type 2 diabetes mellitus (data not shown).

### Colesevelam increases genes involved in de novo bile acid synthesis in microbiome-humanized mice fed a Western diet

**Fig. 4:**
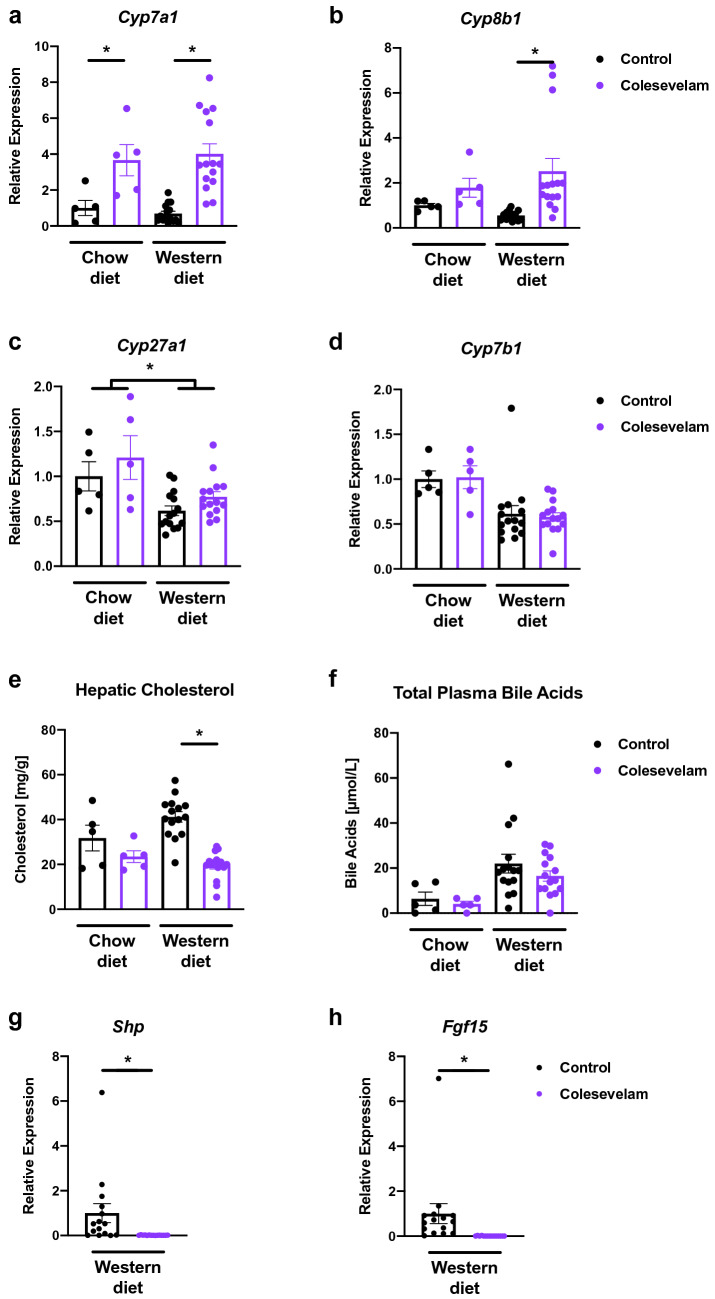
Colesevelam increases expression of hepatic genes involved in de novo bile acid synthesis in Western diet-fed microbiome-humanized mice. Microbiome-humanized C57BL/6 mice were fed a chow diet (*n* = 4–5) or Western diet (*n* = 15) with or without colesevelam for 20 weeks. **a**–**d** Hepatic mRNA expression of **a** *Cyp7a1*, **b** *Cyp8b1*, **c** *Cyp27a1*, and **d** *Cyp7b1*. **e** Hepatic cholesterol content. **f** Total plasma bile acids. **g**–**h** Intestinal mRNA expression of **g** *Shp*, **h** *Fgf15.* Results expressed as mean ± s.e.m. *p* values are determined by Mann–Whitney test **a**–**b**, **d**–**h** or two-way ANOVA **c** with Holm’s post hoc test. *p< 0.05. *Cyp7a1,* cholesterol 7α-hydroxylase; *Cyp7b1,* oxysterol 7α-hydroxylase; *Cyp8b1,* sterol 12α-hydroxylase; *Cyp27a1,* sterol 27-hydroxylase; *Fgf*, fibroblast growth factor; *Shp,* small heterodimer partner

Colesevelam induced enzymes of the classic bile acid synthesis pathway, including the rate-limiting enzymes cytochrome P450 7a1 (*Cyp7a1*) and 8b1 (*Cyp8b1*), after 20 weeks of Western diet feeding compared with mice not given colesevelam (Fig. 4A–B). However, treatment with the bile acid sequestrant did not change mRNA expression of the enzymes of the alternative bile acid synthesis pathway cytochrome P450 27a1 (*Cyp27a1*) and 7b1 (*Cyp7b1*) (Fig. 4C–D). Hepatic cholesterol, the substrate for de novo bile acid synthesis, was reduced in mice administered colesevelam in relation to their controls after 20 weeks of Western diet feeding (Fig. 4E). The sequestrant did not alter total plasma bile acid levels after Western diet or chow diet feeding over 20 weeks, although the former group had significantly higher levels than the latter (Fig. 4F). Bile acid sequestrants are known to decrease intestinal farnesoid X receptor (Fxr) signaling by luminal binding of bile acids, which modulates hepatic *Cyp7a1* expression [14]. We, therefore, assessed the intestinal gene expression of Fxr-dependent genes small heterodimer partner (*Shp*) and fibroblast growth factor 15 (*Fgf15*), which were both significantly decreased in colesevelam-treated mice in relation to their Western diet-fed counterparts not treated with the bile acid binder (Fig. 4G–H).

### Colesevelam decreases plasma lipopolysaccharide (LPS) levels and induces the gene expression of intestinal antimicrobials in microbiome-humanized mice fed a Western diet

**Fig. 5:**
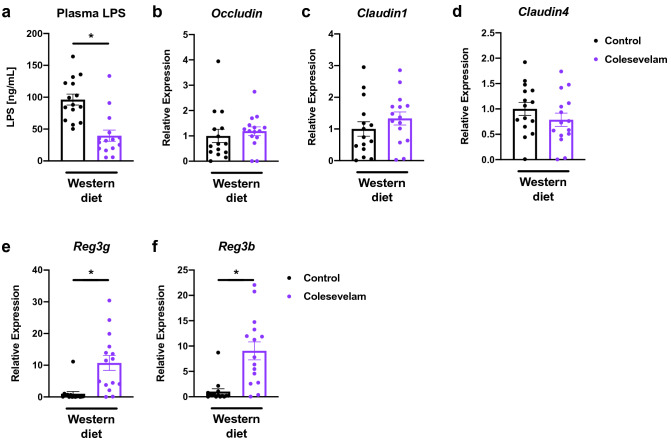
Colesevelam reduces LPS plasma levels and increases intestinal expression of antimicrobial genes in Western diet-fed microbiome-humanized mice. Microbiome-humanized C57BL/6 mice were fed a Western diet (*n* = 15) with or without colesevelam for 20 weeks. **a** Plasma LPS levels. **b**–**h** Distal small intestinal mRNA expression of **b** *Occludin*, **c** *Claudin 1*, **d** *Claudin 4*, **e** *Reg3g*, and **f** *Reg3b*. Results expressed as mean ± s.e.m. *p* values are determined by Mann–Whitney test **a**–**b**, **e**–**f** or unpaired Student’s *t* test **c**–**d**. *p < 0.05. *LPS*, lipopolysaccharides; *Reg3*, regenerating islet-derived protein 3

LPS is known to contribute to the development of liver steatosis, inflammation, and fibrosis in rodent models of diet-induced steatohepatitis [15, 16]. We, therefore, quantitated the LPS plasma levels in our mouse model, and found that colesevelam significantly decreased LPS concentrations in Western diet-fed mice (Fig. 5A). To explain the difference in LPS levels, we next determined the gene expression of major intestinal tight junction proteins, but did not detect significant differences in the expression of *Occludin*, *Claudin 1*, and *Claudin 4* between Western diet-fed mice treated with colesevelam and those not treated with the bile acid sequestrant (Fig. 5B–D). We then assessed intestinal antimicrobials, and found that colesevelam treatment was associated with an increased gene expression of the antimicrobials regenerating islet-derived protein 3 gamma and beta (*Reg3g* and *Reg3b*) in Western diet-fed mice (Fig. 5E–F).

## Discussion

NAFLD and NASH are leading causes of morbidity, liver cirrhosis, and liver transplantation [2–4]. Here, we demonstrate that colesevelam decreases Western diet-induced weight gain, hepatic inflammation, steatosis, and fibrosis, and insulin resistance in mice colonized with stool from patients with NASH. This was associated with increased expression of de novo bile acid synthesis enzymes, lower plasma LPS levels, and increased intestinal expression of the antimicrobials *Reg3g* and *Reg3b*.

We have shown that both alcohol-induced and diet-induced liver disease are associated with bile acid dysregulation in mice [17, 18]. We have also demonstrated that modulation of the farnesoid X receptor (Fxr)-fibroblast growth factor 15/19 (Fgf15/19)-Cyp7a1 axis with the intestinal Fxr-agonist Fexaramine and adeno-associated virus-Fgf19-variants improves both conditions [17, 18].

In the present study, we demonstrate that colesevelam reduces Western diet-induced obesity and associated metabolic dysregulation in mice, as indicated by a significantly lower body weight gain and better insulin response compared with control mice that do not receive the bile acid binder. It has been shown that colesevelam improves metabolic responses, such as lowering hepatic glucose production by suppressing hepatic glycogenolysis as well as increasing insulin sensitivity, in diet-induced obese rodents by activation of the intestinal Takeda G protein-coupled receptor-5 (Tgr-5) and glucagon-like peptide-1 (Glp-1) release [7, 19]. These beneficial metabolic effects are consistent with studies in humans, where 12 and 24 weeks of treatment with colesevelam significantly improved hemoglobin A1c and low-density lipoprotein-cholesterol in patients with type 2 diabetes mellitus [9, 10].

Further, we show that colesevelam reduces liver weight and hepatic steatosis after 20 weeks of Western diet feeding relative to controls. This is in contrast to a study in patients with NASH, which showed that treatment with 3.75 g of colesevelam daily over 24 weeks resulted in significant worsening of liver fat per magnetic resonance imaging-proton-density-fat-fraction (MRI-PDFF) as well as conventional magnetic resonance spectroscopy (MRS) [20]. Liver biopsy on the other hand did not detect any effect of treatment [20]. Contrary to these findings, treatment with another bile acid sequestrant Colestimide (3 g daily) over 24 weeks resulted in a significant improvement of hepatic steatosis and visceral fat in patients with NASH [21]. It is unclear how colesevelam deteriorated liver fat content in the prior study given several studies demonstrating beneficial metabolic effects of bile acid sequestrants and colesevelam in particular in rodent and human studies. Colesevelam was also shown to be protective in other experimental liver diseases such as cholestatic liver disease [22] or ethanol-induced liver disease in rodents [23]. Moreover, the bile acid sequestrant sevelamer was found to improve diet-induced steatohepatitis in rodents as well [14, 24], whereas another bile acid binder, cholestyramine, given over 8 weeks did not improve hepatic steatosis in ob/ob mice [25]. The difference in the hepatic phenotype could possibly be explained by a difference in treatment duration (e.g., 20 weeks as in our model versus 8 weeks in the ob/ob mouse model [25]). It could be that a significant depletion of hepatic cholesterol secondary to bile acid sequestrant use is required – as in our study – to achieve improvement of diet-induced steatohepatitis [26]. Similarly, this could explain why in the human studies above colesevelam deteriorated [20] whereas colestimide improved human NASH [21]. Although both bile acid binders were administered over 24 weeks, it could be that a longer treatment duration with colesevelam would have been necessary to result in depletion of hepatic cholesterol with an associated possible amelioration of NASH [26]. Further, there are genetic, metabolic, and microbiome-related differences between rodents and humans, which could partially account for the different effects between the species. Additionally, it is important to note that metabolism is different between different liver diseases, disease stages, and associated other conditions including metabolic syndrome or diabetes mellitus. The inconsistent and sometimes contradictory results in preclinical and clinical studies about bile acid sequestrants in NASH could explain the lack of ongoing clinical trials of bile acid binders in NASH.

Colesevelam was previously found to suppress ileal Fgf15 expression due to its binding of bile acids, which subsequently derepresses hepatic Cyp7a1 expression via the Fxr–Fgf15–Cyp7a1 axis in a mouse model of high-fat diet-induced obesity [7], which we confirmed in our microbiome-humanized mice. Furthermore, it induces the other enzyme of the classic de novo bile acid synthesis pathway Cyp8b1 in relation to the Western diet-fed control mice. Conversely, the enzymes of the alternative/acidic bile acid synthesis pathway are not induced by colesevelam compared with Western diet-fed controls. The main primary bile acids synthesized via the hepatic de novo bile acid synthesis pathways are cholic acid and chenodeoxycholic acid; their ratio is determined by the expression of Cyp8b1 [27]. The increased expression of Cyp8b1 in the colesevelam group relative to its Western diet-fed counterpart not treated with colesevelam likely skews the production in favor of cholic acid. This is consistent with studies in humans where colesevelam treatment preferentially increases cholic acid levels but decreases chenodeoxycholic acid levels [28]. Colesevelam binds intestinal bile acids, inhibits the enterohepatic circulation of bile acids and induces conversion of hepatic cholesterol into bile acids [6, 7], which explains the significantly lower hepatic cholesterol content in Western diet-fed colesevelam-treated mice versus Western diet-fed controls despite similar total plasma bile acid levels. This liver cholesterol lowering effect is likely very important for the protection against diet-induced liver disease [26]. Additionally, we demonstrated that colesevelam decreases plasma LPS levels in Western diet-fed mice, which might at least partially be the result of increased intestinal expression of antimicrobial proteins. Furthermore, colesevelam likely reduces plasma LPS levels by directly binding intestinal LPS, as bile acid sequestrants are known to bind luminal LPS in the intestine [24]. As LPS contributes to the development of liver steatosis, inflammation, and fibrosis in diet-induced steatohepatitis [15, 16], this LPS lowering effect represents another mechanism how colesevelam improves diet-induced liver disease.

## Conclusion

In conclusion, colesevelam decreases Western diet-induced weight gain, hepatic inflammation, steatosis, and fibrosis, and insulin resistance in mice colonized with stool from patients with NASH, indicating a potential benefit of bile acid sequestration in obesity and NASH. However, larger clinical trials with bile acid sequestrants are required to better evaluate therapeutic effects in patients with obesity and NASH.

## Data Availability

The data that support the findings of this study are available from the corresponding author BS upon request.

## Abbreviations

Adgre1: Adhesion G protein-coupled receptor E1
Ccl2: Chemokine C–C motif ligand-2
Col1a1: Collagen type I alpha 1
Cyp: Cytochrome P450
Fgf: Fibroblast growth factor
Fxr: Farnesoid X receptor
LB: Luria–Bertani
LPS: Lipopolysaccharides
MRI-PDFF: Magnetic resonance imaging-proton-density-fat-fraction
MRS: Magnetic resonance spectroscopy
NAFLD: Non-alcoholic fatty liver disease
NASH: Non-alcoholic steatohepatitis
Shp: Small heterodimer partner
Reg3: Regenerating islet-derived protein 3
Tgfb1: Transforming growth factor-beta 1
Tgr-5: Takeda G protein-coupled receptor-5
Glp-1: Glucagon-like peptide-1
Timp1: Tissue inhibitor of metalloproteinase-1
Tnf-α: Tumor necrosis factor-alpha
